# Deciphering Seed Deterioration: Molecular Insights and Priming Strategies for Revitalizing Aged Seeds

**DOI:** 10.3390/plants14111730

**Published:** 2025-06-05

**Authors:** Weigeng Xing, Yi Li, Linyan Zhou, Hao Hong, Yuan Liu, Shuailong Luo, Jialong Zou, Yan Zhao, Yanfei Yang, Zhenjiang Xu, Bin Tan

**Affiliations:** College of Agriculture, Guangzhou Sub-Center for New Plant Variety Tests of the Ministry of Agriculture and Rural Affairs, South China Agricultural University, Guangzhou 510642, China; 20233137098@stu.scau.edu.cn (W.X.); 16607341890@163.com (Y.L.); zhoulinyan00@163.com (L.Z.); honghao0222@163.com (H.H.); 15665152059@163.com (Y.L.); 13767082873@163.com (S.L.); zjl16635048919@163.com (J.Z.); zhaoyan09122022@163.com (Y.Z.); yyfwork1028@163.com (Y.Y.)

**Keywords:** seed deterioration, seed storage longevity, seed priming, mechanism

## Abstract

Seed deterioration is an inevitable process during storage, characterized by a gradual loss of germination capacity and eventual seed death, which poses challenges to seed longevity and the preservation of genetic resources. Understanding the molecular mechanisms driving seed aging and inherent resistance pathways, alongside developing innovative rejuvenation strategies for deteriorated seeds, is crucial for agricultural sustainability and germplasm banking. This review systematically examines (1) redox-regulated deterioration pathways involving reactive oxygen species (ROS) and macromolecular damage cascades, (2) anti-deterioration mechanisms mediated by the antioxidant system and macromolecular repair mechanisms, (3) genetic–epigenetic networks governing seed aging resistance, particularly ABA- and IAA-mediated signaling through ABI3/ABI5/LEC1 regulons, and (4) technological advances in seed priming that restore aged seeds via metabolic resetting and repair potentiation. By integrating multi-omics insights with physiological evidence, we propose a hierarchical model of seed deterioration and establish mechanistic links between priming interventions and longevity enhancement. These insights offer a theoretical framework for cultivating anti-deterioration crop varieties and developing seed longevity-enhancement technologies.

## 1. Introduction

Seeds, as the fundamental reproductive units of plants, play a pivotal role in the evolution and survival of higher plants [1]. Maintaining germination capacity serves as the foundation for fulfilling their reproductive function. Seed germination capacity peaks at physiological maturity or when dormant seeds attain maximum viability through after-ripening and/or stratification treatments. However, after this peak, seeds inevitably undergo progressive viability loss until eventual death—a natural process termed seed deterioration or aging. In agricultural production, deteriorated seeds not only exhibit compromised germination performance (e.g., delayed germination and reduced uniformity) but also impair seedling development through low establishment rates, uneven growth patterns, and heightened susceptibility to abiotic/biotic stresses [2]. Consequently, seed deterioration directly undermines reproductive success and diminishes agricultural value.

Although seed deterioration is inevitable, the rate of the process is determined by both intrinsic seed properties and extrinsic environmental factors. Seed deterioration is intricately associated with morphological, physiological, and biochemical alterations in stored seeds, including compromised seed coat structure, excessive reactive oxygen species (ROS) generation, lipid peroxidation, depletion of storage reserves, aberrant protein modifications, dysregulated gene expression and accumulated genetic damage [3]. Storage conditions—particularly ambient temperature, relative humidity, atmospheric composition, and microbial activity—are critical determinants of deterioration kinetics [4,5,6].

Current strategies to delay deterioration (e.g., low-temperature preservation, ultra-dry storage, anaerobic storage) focus on environmental modulation [4,5,6], yet these methods merely decelerate aging and prove ineffective for already deteriorated or low-vigor seeds. In contrast, seed priming has emerged as a robust intervention that reactivates repair mechanisms (e.g., DNA damage repair, protein refolding) and potentiates antioxidant responses in aged seeds [7]. Priming treatment significantly enhances germination synchrony, improves seedling stress tolerance and disease resistance, and elevates crop yield with enriched grain micronutrient profiles [8,9,10]. Notably, its efficacy in revitalizing deteriorated seeds has been validated across staple crops like maize and rice [11,12].

## 2. Determinants of Seed Deterioration

### 2.1. External Factors

Seed storage longevity is critically influenced by storage conditions, including temperature, relative humidity (RH), environmental gas composition, and biotic factors [13]. Based on desiccation sensitivity, seeds are categorized into three types: orthodox, intermediate, and recalcitrant [14]. Orthodox seeds can be desiccated to internal moisture contents below 12% (on a fresh weight basis) and survive long-term storage at subzero temperatures. Recalcitrant seeds cannot be stored in a conventional freezer, as they are unable to survive after drying and/or freezing at −20 °C. Intermediate seeds tend to age faster than orthodox seeds and may have a lifespan of only 5 years when stored at −20 °C. They achieve their greatest longevity when dried to equilibrium with 45–65% RH [15]. Notably, orthodox seeds are the most prevalent, particularly among cultivated plants, and have been the most extensively studied.

For orthodox seeds, seed moisture content (or equilibrium RH) and temperature are key factors influencing seed storage longevity. Based on the constant relationships among storage temperature, moisture content, and longevity, several general rules for seed storage have been proposed. Two simple and commonly applied rules include (1) James’ Rule: Ideal storage conditions require that the temperature (°F) plus the ambient RH (%) remain below 100. For example, at 50% RH, the storage temperature should be below 50 °F (10 °C) to achieve medium-term seed storage; (2) Harrington’s Rule: Within temperature ranges of 0–40 °C and moisture content ranges of 5–14%, the seed storage longevity approximately doubles for every 10 °F (5.6 °C) reduction in temperature and every 1% reduction in seed moisture content. Both rules emphasize the critical importance of low seed moisture content and low temperature for prolonging seed longevity, particularly highlighting the need to avoid the synergistic effects of high temperature and high moisture content [16]. It is noteworthy that the relationships among storage temperature, moisture content, and longevity are not necessarily constant or even linear at the scales on which these properties are measured. Based on the observed patterns of crop seed longevity under varying temperature and moisture conditions during storage, Ellis & Roberts developed a predictive log-linear regression equation for orthodox seeds using statistical methodologies [17,18]. This equation provides a more accurate representation of the quantitative relationships among storage temperature, seed moisture content, and longevity, indicating that longevity increases as both storage temperature and moisture content decrease [19,20]. Therefore, to facilitate research on seed longevity, accelerated seed deterioration (artificial aging, AA) methods have been developed. These techniques utilize high-temperature and high-humidity conditions to accelerate the deterioration process and shorten seed storage longevity for experimental purposes.

For recalcitrant and intermediate seeds, cryopreservation technologies represent the most efficient method to preserve viability. Cryopreservation involves storage at ultralow temperatures, often in liquid nitrogen (−196 °C). Rapidly advancing methods can be used to essentially stop water from freezing within recalcitrant seed cells and obviate lethal freezing damage. The technology requires careful dehydration of tissues and extremely rapid cooling [21].

RH serves as the primary driver of deterioration by mediating enzymatic reactivation and non-enzymatic oxidation. Elevated seed moisture accelerates deterioration through oxidative modifications (e.g., lipid peroxidation, protein carbonylation, DNA strand breaks) and microbial proliferation [22]. Under desiccated conditions (<5% moisture), molecular oxygen interacts with intracellular transition metals (e.g., Fe^2+^, Cu^+^) via Fenton/Haber–Weiss reactions, generating hydroxyl radicals (•OH) that oxidize macromolecules, thereby exacerbating cellular dysfunction [22].

The gas composition of seed storage containers has received considerable attention for its role in modulating longevity. Oxygen is hypothesized to accelerate seed deterioration through oxidative reactions. In general, compared to storage in air, seed survival is prolonged in nitrogen (N_2_), carbon dioxide (CO_2_), or vacuum environments, whereas elevated oxygen partial pressures accelerate the loss of viability [16].

Biotic stressors—including rodents (*Rattus* spp.), insects (*Sitophilus zeamais*), and fungal pathogens (*Aspergillus* spp.)—threaten stored seeds through distinct mechanisms: rodents and insects cause physical damage via consumption, while pathogens secrete hydrolases to degrade storage reserves (e.g., starch, lipids), depleting germination energy and inducing mycotoxin-mediated deterioration [23].

Additionally, seed processing technologies, including seed harvesting, drying, cleaning, packaging, and transportation, influence seed deterioration. For instance, mechanical injuries incurred during these processes not only directly accelerate seed aging but also enhance microbial invasion risks by compromising seed coat integrity. Furthermore, excessive, or overly rapid drying rates can compromise embryo cell integrity through membrane lipid peroxidation and protein denaturation, while insufficient drying predisposes seeds to metabolic reactivation and fungal proliferation.

### 2.2. Internal Factors

The seed coat (*testa*) serves as a critical protective barrier between the embryo and the external environment, regulating water and gas exchange primarily through the hilum—a specialized structure that facilitates photosynthetic product transport to the developing embryo during seed maturation. The structural integrity of the hilum directly modulates seed moisture content, with high-moisture seeds exhibiting accelerated deterioration rates due to enhanced enzymatic and oxidative reactions, rendering them unsuitable for long-term storage. Compromised seed coat integrity predisposes seeds to microbial colonization and accelerates deterioration by promoting premature mobilization of storage reserves (e.g., lipid hydrolysis, starch degradation). Hydrophobic substances in the seed coat, such as cuticular wax—a hydrophobic barrier synthesized during late seed maturation—restrict external water infiltration through the *testa*, thereby maintaining low seed moisture content at levels optimal for storage stability. Furthermore, the physical characteristics of the seed coat, including cuticular cracks, micropyle aperture size, and *testa*-cotyledon interfacial gaps, collectively govern seed permeability, biotic stress resilience, and long-term storage potential [24,25]. In summary, these factors collectively influence the protective functions of the seed coat, thereby regulating internal physiological activities within the seed.

Seed chemical composition significantly influences deterioration dynamics, particularly through the content and composition of storage reserves. Oils are prone to oxidation and rancidity, generating harmful aldehydes (e.g., malondialdehyde) and free fatty acids that induce cellular damage via membrane lipid peroxidation. Comparative studies reveal that low-lipid seeds (<10% lipid content) exhibit extended longevity compared to high-lipid counterparts (>20%), attributed to reduced oxidative substrate availability [26]. Non-storage reserve components—including chlorophyll, polysaccharides, and phenolic compounds—also modulate deterioration rates. Chlorophyll-retaining seeds demonstrate accelerated aging due to photooxidative stress from retained chloroplasts, which generate singlet oxygen (^1^O_2_) under light exposure [27,28]. Conversely, phenolic antioxidants like flavonoids mitigate oxidative damage by scavenging ROS and chelating transition metals.

The physiological state of seeds influences seed deterioration. During seed development, the dry weight of seeds increases significantly, reaching its maximum at the end of the grain-filling phase when seeds attain physiological maturity and their germination capacity peaks [29,30,31]. Premature harvest (before physiological maturity) often results in underdeveloped seed structures (e.g., incomplete *testa* lignification) and insufficient accumulation of storage reserves (e.g., proteins, lipids), leading to reduced germination rates and increased susceptibility to oxidative deterioration. Conversely, delayed harvest exposes seeds to field risks such as fungal infection (*Aspergillus* spp.), mechanical damage (e.g., threshing-induced fractures), and desiccation-induced fissures, which collectively contribute to poor germination performance, accelerated aging, and shortened storage longevity [32]. Therefore, seed maturity not only determines the initial germination vigor of stored seeds but also modulates their deterioration kinetics during storage.

The intracellular biochemical reactions of seeds critically influence seed deterioration. Two predominant hypotheses explain the relationship between embryonic cell biochemistry and aging: (1) Membrane lipid peroxidation hypothesis: Free radicals generated through autoxidation or enzymatic oxidation in seeds initiate oxidative attacks on polyunsaturated fatty acids (PUFAs) in membrane lipids. This process generates lipid hydroperoxides (LOOHs) that compromise plasma membrane integrity (e.g., increased electrolyte leakage), thereby accelerating deterioration [2]; (2) Mitochondrial theory of aging: In seed cells lacking chloroplasts, mitochondrial electron transport chains (ETCs) become the primary source of ROS (e.g., superoxide anion, H_2_O_2_). Cumulative ROS accumulation induces oxidative damage to mitochondrial macromolecules—including mtDNA deletions, cytochrome C oxidase inactivation, and cardiolipin peroxidation—which disrupts energy metabolism and accelerates senescence [33].

The genetic background of seeds constitutes the fundamental regulator of deterioration processes, as it governs seed structure, chemical composition, physiological state, and biochemical reactions. Hormonal signaling pathways—particularly abscisic acid (ABA) and indole-3-acetic acid (IAA)—orchestrate seed development and maturation by modulating expression of key regulatory genes (e.g., *TRANSPARENT TESTA* family for seed coat development), controlling enzymatic activity in secondary metabolism (e.g., chalcone synthase for flavonoid biosynthesis), and activating storage compound synthesis genes (e.g., *oleosins* for lipid body formation). Concurrently, molecular chaperones (heat shock proteins [HSPs]) and late embryogenesis abundant proteins (LEAs) are synthesized during seed development to stabilize proteomes and enhance desiccation tolerance, while antioxidant enzymes (e.g., superoxide dismutase [SOD], catalase [CAT]) are produced to bolster redox homeostasis [34]. These intrinsic factors collectively determine seed deterioration susceptibility through integrated genetic, biochemical, and biophysical mechanisms.

## 3. Morphological, Anatomical, and Physiological-Biochemical Changes in Aged Seeds

### 3.1. Morphological Changes

Seeds undergo significant morphological changes during storage. For example, rice seeds develop reddish husk pigmentation after long-term storage [1]. Structural modifications also occur internally with prolonged storage duration. Freshly harvested lentil (*Lens culinaris*) seeds exhibit olive-green cotyledons that gradually fade to light yellow and eventually turn dark brown after two years of storage, a phenomenon attributed to chlorophyll degradation in their cotyledons [35]. In watermelon (*Citrullus lanatus*) and melon (*Cucumis melo* L.) seeds stored for five years, non-viable seeds show pronounced separation between the seed coat and embryo. These deteriorated embryos appear shrunken and irregularly shaped, with structural alterations resulting from substrate depletion and cellular damage [36]. Therefore, the seed coat’s protective capacity becomes compromised.

### 3.2. Ultrastructural Changes

During the process of seed deterioration, ultrastructural changes occur in the organelles of embryo cells, including mitochondria, endoplasmic reticulum, and Golgi apparatus. Mitochondria, as the most important organelles for cellular energy metabolism, serve as the primary source of ROS. Seed deterioration is closely related to mitochondrial degradation. In this process, mitochondria in aged embryo cells become significantly enlarged and exhibit distorted outer membranes, while cristae become disorganized or even absent. These structurally compromised mitochondria render cells incapable of carrying out respiratory metabolism and producing ATP required for life activities, ultimately leading to cell dysfunction and apoptosis [37].

The Golgi apparatus and endoplasmic reticulum, which are critical sites for cell metabolism and protein synthesis, also undergo fragmentation or degradation in deteriorated embryo cells. Concurrently, lipid droplets fuse to form larger irregular bodies. These alterations disrupt material metabolism and protein synthesis, resulting in cellular dysfunction. Other ultrastructural changes include the dissolution of membrane-bound structures (e.g., protein bodies, vacuoles, and plasma membranes), cell wall contraction, and nuclear chromatin condensation with lobulation [38]. Collectively, these pathological changes induce cell death and progressive loss of embryo viability.

### 3.3. Physiological and Biochemical Changes

**Figure 1:**
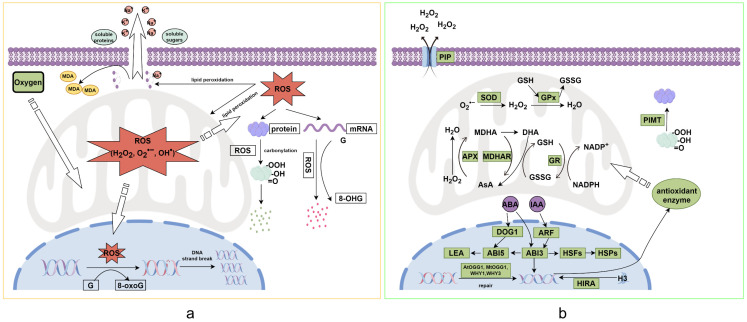
Mechanisms of Seed Deterioration (**a**) and Anti-Deterioration (**b**) (By Figdraw 2.0). (**a**) Cellular Oxidative Damage: Oxygen molecules entering mitochondria undergo respiratory metabolism, concurrently generating reactive oxygen species (ROS: H_2_O_2_, O_2_^·−^, OH^·^). These ROS mediate multilevel oxidative damage: (i) In the nucleus, ROS oxidize guanine (G) to 8-oxo-7,8-dihydroguanine (8-oxoG), inducing DNA strand breaks and replication errors; (ii) In the cytoplasm, ROS catalyzes protein carbonylation through metal-catalyzed oxidation, rendering modified proteins prone to ubiquitin-proteasomal degradation; (iii) Cytosolic mRNA undergoes guanine oxidation to 8-hydroxyguanosine (8-OHG), destabilizing transcript integrity; (iv) Membrane phospholipids undergo peroxidation chain reactions, generating malondialdehyde (MDA) as a byproduct while compromising membrane semi-permeability, leading to electrolyte leakage and organelle dysfunction. (**b**) Cellular Anti-Deterioration Mechanisms: Phytohormone signaling networks (e.g., ABA, IAA) orchestrate counteractive responses by (i) Upregulating DNA repair enzymes (e.g., OGG1 glycosylase) and antioxidant genes (e.g., SOD, CAT, APX), enhancing ROS-scavenging capacity via mitochondrial Mn-SOD activation; (ii) Protein-L-isoaspartyl methyltransferase (PIMT) catalyzes the repair of isoaspartyl residues in damaged proteins through methylation-demethylation cycles, restoring structural conformation; (iii) Aquaporin-mediated ROS efflux channels facilitate H_2_O_2_ extrusion across plasma membranes, while glutathione-ascorbate redox cycles maintain cytoplasmic redox homeostasis. Abbreviations: 8-OHG: 8-hydroxyguanosine, 8-oxoG: 8-oxoguanine, ABA: abscisic acid, APX: ascorbate peroxidase, AsA: ascorbic acid, CAT: catalase, GPx: glutathione peroxidase, GR: glutathione reductase, GSH: glutathione, HSP: heat shock protein, IAA: auxin, LEA: late embryogenesis abundant protein, MDA: malondialdehyde, PIP: plasma membrane intrinsic protein, RFO: raffinose family oligosaccharide, ROS: reactive oxygen species, SOD: superoxide dismutase. The black arrow indicates the activation reaction, and the white wide arrow indicates the substance transportation.

The physiological and biochemical changes associated with seed deterioration primarily include oxidative damage, stored-reserve consumption, and changes in plant hormone levels (Figure 1a). ROS chemically reacts with antioxidant enzymes, structural proteins, and genetic material, leading to the inactivation, instability, or even degradation of these substances. Damage to the antioxidant enzyme system arises from an imbalance between oxidants and antioxidants, with excessive ROS accumulation in mitochondria disrupting redox homeostasis [39]. In pigeon pea (*Cajanus cajan*) seeds subjected to accelerated deterioration treatment, the activities of SOD, CAT, and ascorbate peroxidase (APX) are significantly reduced [40]. Similarly, aged black pine (*Pinus thunbergia*) seeds exhibit diminished SOD, glutathione reductase (GR), and CAT activities, severely compromising their antioxidant defense system [41].

ROS are the primary cause of DNA damage in aged seeds and can induce the hydroxylation modification of the eighth carbon of guanine, resulting in 8-oxoguanine (8-oxoG), which is the most common form of guanine modification and is highly prone to causing base mispairing and DNA single-strand breaks [42]. The accumulation of DNA damage in aged seeds leads to an increased frequency of chromosomal structural variations, such as the formation of bridges due to the chromosomal breakage-fusion-bridge cycle [43,44]. Due to the lack of specialized repair mechanisms, RNA is more sensitive to oxidative damage; the oxidation modification of guanine results in 8-hydroxyguanosine (8-OHG), which reduces mRNA stability and causes codon mismatch during translation, thereby affecting protein synthesis [45]. Oxidative damage to mRNA can cause the fragmentation observed in soybean seeds over 20 years of storage [46,47]. Previous studies have shown that seeds accumulate mRNAs during late seed maturation. Unlike the majority of RNAs in metabolically active cells, seed-stored mRNAs are long-lived, being able to survive for many years [40,41]. These long-lived mRNAs display the ability to survive desiccation and remain translatable in dry quiescent embryos. For seeds to germinate, translation of seed-stored mRNA into proteins is essential. The stored mRNA in dry seeds can be immediately utilized for translation to proteins required by the embryo cells in the pre-germination stage [48]. However, due to the poor stability of mRNA, it gradually becomes damaged and degraded during seed storage, which in turn affects the embryo cells’ vital activities. Therefore, the total amount and integrity of stored mRNA in seeds are positively correlated with seed longevity and can serve as predictive factors for the degree of deterioration in dried stored seeds [49,50,51,52].

Lipid peroxidation is an initial event in the seed deterioration process and is closely associated with the loss of seed viability [53]. Malondialdehyde (MDA), the product of lipid peroxidation, reflects the degree of oxidative damage to membranes and their integrity. Thus, MDA serves as a critical biochemical marker for evaluating seed deterioration progression [54]. Compared to freshly harvested beech (*Fagus sylvatica*) seeds, the MDA content in 17-year-stored seeds significantly increased 5–6-fold in cotyledons and hypocotyls [55]. Similarly, MDA levels in naturally aged (NA) and AA seeds of physic nut (*Jatropha curcas*) were significantly higher than in fresh seeds [56]. Lipid peroxidation disrupts plasma membrane integrity, leading to leakage of intracellular solutes (primarily charged ions like Na^+^ and K^+^). Therefore, the electrical conductivity (EC) of seed-soaking solutions correlates with membrane damage severity [57]. After 14 days of AA treatment, elm (*Ulmus parvifolia*) seeds showed significantly reduced germination ability and higher EC compared to untreated controls [58]. The EC of physic nut seed soaking solutions also significantly increased after NA and AA treatments [56].

ROS generated during the seed deterioration process can enter protein carbonylation reactions, affecting their function [37]. ROS-mediated protein damage primarily encompasses three categories: (1) direct metal-catalyzed oxidation of sulfur-containing amino acid residues. For example, cysteine (Cys) forms cystine (wherein two molecules of cysteine are linked by a disulfide bond), which can undergo further oxidation to cysteine sulfinic acid and, irreversibly, to cysteic acid [59,60,61,62]; (2) methionine (Met) oxidation, in which Met is converted to methionine sulfoxide (a reversible modification) and subsequently to methionine sulfone, an irreversible product [59]; (3) carbonylation reactions involving amino acid side chains—particularly those of arginine, lysine, proline, and threonine—which yield stable aldehydes or ketones as a result of oxidative modifications [59,63,64].

The storage reserves are fundamental to the formation of seed viability and are also significant factors for the preservation of seed viability during storage. During the seed storage phase, the content of carbohydrates, proteins, and lipids within seeds tends to decrease, leading to seed deterioration and a decline in vitality. Soluble sugars, which serve as the primary respiratory substrates, are progressively consumed during storage. For example, the contents of fructose and glucose in brazilwood (*Caesalpinia echinata*) seeds stored at room temperature decreased by 71.7% and 72.2%, respectively [65]. Under both NA and AA treatments, oligosaccharide content in the hypocotyls and cotyledons of *Melanoxylon brauna* seeds significantly decreased, while glucose content increased [66]. Proteomic analyses have revealed that soluble protein content markedly declines during seed deterioration, accompanied by alterations in protein composition [67,68]. The oxidation and carbonylation of proteins during deterioration reduce enzymatic activity or induce functional loss, thereby impairing metabolic processes in embryonic and storage tissues. Notably, some studies have demonstrated that seed deterioration enhances intracellular proteolytic enzyme activity, promoting the hydrolysis of storage proteins and accelerating the depletion of reserves, which further exacerbates the deterioration process [69].

## 4. Seed Anti-Deterioration Mechanisms

Seeds have evolved complex anti-deterioration systems to slow down the deterioration process and repair damage caused by deterioration. The seed anti-deterioration system primarily consists of protective systems, repair systems, and detoxifying agents. The protective system includes the seed coat’s protection of internal structures, antioxidant capacity, and protective proteins; the repair system mainly involves the repair of DNA and RNA, protein damage repair, and membrane system repair; detoxifying agents are primarily responsible for clearing toxic metabolites from the cells, such as ROS scavenging systems, antioxidant enzymes, and vitamin E. The various components of the anti-deterioration system work in coordination and are regulated by upstream plant hormones (Figure 1b).

### 4.1. Protective and Detoxification Mechanisms

There are two types of antioxidant systems present within seeds: one is the enzymatic system, composed of enzymes such as peroxiredoxins (Prxs), CAT, GR, SOD, ascorbate peroxidase, monodehydroascorbate reductase (MDHAR), and glutathione S-transferase (GST); the other is the non-enzymatic system, primarily consisting of ascorbic acid (AsA, also known as vitamin C), glutathione (GSH), α-tocopherol (also known as vitamin E), and carotenoids. The antioxidant protection mechanism depends on the moisture status of the seeds. In dry seeds, the scavenging of ROS mainly occurs through the non-enzymatic system involving vitamin E and GSH, while the antioxidant enzyme system, such as CAT, plays a crucial role in imbibed seeds [70]. Vitamin E is an important antioxidant in seeds, existing in two forms: tocopherols and tocotrienols. Tocopherols protect embryo cells from ROS damage, while tocotrienols may inhibit seed metabolism during deterioration processes. Tocopherols can scavenge lipid peroxide (i.e., free radicals), thereby blocking lipid peroxidation [71,72]. GSH and AsA can protect lipids by converting peroxyl free radicals generated during lipid peroxidation into tocopherol radicals [71,73,74]. Reduced germination rate of aged onion (*Allium cepa* L.) seeds is closely associated with down-regulated expression of genes encoding CAT and SOD, such as *AMY1*, *BMY1*, *CTR1*, and *NPR1* [75]. The expression levels of MDHAR, GR, and GST are significantly downregulated in aged safflower (*Carthamus tinctorius* L.) seeds, leading to excessive accumulation of ROS, and ultimately resulting in loss of seed viability [76].

Reactive nitrogen species (RNS), like ROS, exhibit dual roles in seed physiology. Excessive RNS induces lipid nitration, protein tyrosine nitration, and S-nitrosylation, accelerating seed deterioration [77,78]. RNS is categorized into free radicals (e.g., nitric oxide [NO], nitrogen dioxide [•NO_2_]) and non-radicals (e.g., peroxynitrite [ONOO^−^], nitrosonium [NO^+^]) [79]. In deteriorating seeds, RNS (particularly NO and its derivatives) modulate oxidative stress, mitochondrial function, and posttranslational protein modifications, influencing the progression of seed aging. Whereas, NO mitigates oxidative damage by stimulating glutathione (GSH, reduced glutathione) synthesis in *Arabidopsis* seeds [80], activating ethylene biosynthesis to suppress stress-induced cell death, and enhancing antioxidant enzyme activity (e.g., GR, GPX) in recalcitrant *Antiaris toxicaria* seeds through protein S-nitrosylation/carbonylation balance [81]. Additionally, NO restores metabolic homeostasis in aged apple embryos by elevating phenylalanine and tyrosine levels [82]. The interplay between RNS and ROS, particularly NO and H_2_O_2_, plays a central role in regulating the hypersensitive response—a programmed cell death mechanism critical for pathogen defense. NO fine-tunes ROS production by S-nitrosylating NADPH oxidases (e.g., *AtRBOHD*), while H_2_O_2_ reciprocally stimulates NO synthesis via nitrate reductase activation [83]. These findings highlight NO as a key redox modulator for enhancing seed longevity and stress resilience. However, under specific conditions, the presence of RNS can exacerbate deterioration processes, depending on their concentration and the physiological context.

Antioxidant defense systems reduce ROS-induced protein damage, while chaperone proteins play a similar protective role by interacting with substrate proteins to enhance their stability. This dual mechanism mitigates oxidative stress and maintains proteostasis, thereby preserving seed viability during storage. LEAs, HSPs, and soluble sugars (such as raffinose family oligosaccharides, RFOs) are important components of the protective system and are closely related to dehydration tolerance and seed deterioration. SBP65 is a type of LEA protein; *sbp65* mutants of pea (*Pisum sativum* L.) seeds show short longevity even under dry conditions [84]. In *Arabidopsis*, overexpression of heat stress transcription factors (*HSFs*) enhances the accumulation of HSPs in mature seeds, thereby improving seed vigor [85]. LEAs and HSPs act as chaperones and molecular shields during drying to prevent protein denaturation and membrane instability [86,87]. The synthesis of protective compounds is regulated by plant hormones, for example, ABA regulates the synthesis of HSPs, LEAs, and RFOs [31,88,89,90]. The major transcription factor ABI3 of the ABA signaling pathway directly binds to the promoter of HSFA9, activating its expression and enhancing seed dehydration tolerance [30,91]. ABI5 regulates seed longevity in legumes by controlling RFO synthesis and *LEA* expression levels [92]. The expression of *HSFs* is closely related to auxin signaling, for instance, IAA signaling induces *ABI3* expression, thereby activating *HSFA9* and promoting HSP synthesis, consistent with findings that auxin extends seed longevity [93,94]. The ability of small heat shock proteins (sHSPs) to form large oligomeric complexes underpins their molecular chaperone activity [95]. Therefore, sHSPs enhance the folding of newly synthesized proteins and the refolding of damaged peptides, while combating reactive oxygen species in seeds [28,31,95,96].

### 4.2. Repair Mechanisms

In addition to protective and detoxification mechanisms, repair mechanisms represent another critical strategy for embryonic cells to counteract deterioration, ensuring genomic and proteomic integrity during seed aging and storage. The repair system addresses the damage caused by seed deterioration through a series of biochemical reactions that restore the structural or functional integrity of damaged DNA, RNA, proteins, and membrane systems. ROS induces DNA damage such as base mismatches or double-strand breaks, while DNA integrity is essential for cell division. Seed germination capacity requires intact DNA in embryonic cells to sustain their cell division capability; thus, repairing damaged DNA can enhance the germination ability of deteriorated seeds by restoring mitotic activity in the embryo [97]. OGG1 is a bifunctional DNA glycosylase/AP lyase that removes 8-oxoG through a β-elimination reaction and cleaves DNA at the 3′ side of the resulting AP site. Overexpression of *AtOGG1* reduces DNA damage during the storage-aging process of dry seeds and enhances DNA repair during germination, thereby promoting seed longevity in *Arabidopsis* [42]. The expression of *MtOGG1* is involved in oxidative stress responses during seed imbibition, activating DNA repair to counteract ROS damage [98]. The DNA-binding protein family WHIRLY (WHY) plays a crucial role in maintaining genomic stability in both the nucleus and organelles. Mutants of *why1* and *why3* exhibit reduced longevity after deterioration compared to wild-type plants, and the double mutant *why1why3* is highly sensitive to deterioration in *Arabidopsis* [99].

There is a close relationship between protein repair and seed longevity [100]. L-isoaspartate methyltransferase (PIMT), an S-adenosylmethionine (SAM)-dependent enzyme, converts abnormal L-isoaspartate residues (isoAsp) into the typical L-aspartate form (Asp). Overexpression of PIMT in rice and chickpea seeds enhances seed viability and longevity [101,102].

## 5. Genetic Regulation of Seed Deterioration

Many genes regulating the seed deterioration process have been identified in different plants. Some of these genes influence seed deterioration by regulating various stress proteins (such as LEAs and HSPs), while others interact to regulate dehydration tolerance and dormancy in deteriorating seeds. *ABI3*, *ABI5*, and *LEC1* form a synergistic transcriptional regulatory network, where *LEC1* acts as an upstream regulator that directly activates the expression of *ABI3* and *ABI5*. Together, they integrate abscisic acid (ABA) signaling with embryo development programs to control seed maturation, dormancy, and storage protein synthesis (e.g., 2S albumin and CRA1) [103]. Furthermore, they enhance coordinated repression of downstream stress-responsive genes through protein–protein interactions, ensuring precise temporal control of germination timing. Additionally, some genes improve the antioxidant enzyme system’s activity, thereby mitigating seed deterioration. Table 1 summarizes the reported regulatory genes and their effects on seed deterioration and longevity.

Epigenetics involves the control of gene expression without altering the DNA sequence, primarily including DNA methylation, histone modifications, and microRNAs. Cytosine methylation plays a crucial role in regulating gene expression, and DNA methylation mediates plant responses to environmental stresses. In seeds stored for up to three years, a significant influence of storage time on DNA methylation was observed. DNA methylation, especially the level of 5-methylcytosine (m5C) in seeds, can be used as a truly universal viability marker regardless of the postharvest category of seeds and their composition. Maintaining high levels of DNA methylation delays seed deterioration and extends longevity [104]. Histones, as nucleosome core components, stabilize chromatin structure and maintain genome integrity. In *Arabidopsis*, mutations in the *HISTONE REGULATOR A* (*HIRA*) gene reduce H3 histone levels and increase chromatin accessibility, hence *hira* mutant seeds exhibit increased dormancy and shortened lifespan after aging treatment, likely due to accumulated DNA damage [105]. In *Arabidopsis* histone H3.3 plays a significant role in coordinating the expression of genes involved in seed maturation and germination. This protein binds the 5′ region of related genes and opens the DNA helix to make it available for expression. On the other hand, it has also an affinity for the 3′ end of related genes, thereby preventing their improper expression, i.e., cryptic transcription starts sites. The latter function is achieved by stimulating DNA methylation at the end of the gene body. So, the loss of H3.3 results in severely impaired germination and post-embryonic development [106]. Small RNAs, including microRNAs (*miRNAs*) and small interfering RNAs (*siRNAs*), post-transcriptionally regulate stress-responsive genes. Downregulation of *miR164*, *miR6260*, *miR5929*, *miR6214*, *miR3946*, and *miR3446* activates stress responses and antioxidant systems, enhancing seed viability [107].

In summary, epigenetic regulation is pivotal in the seed deterioration process. However, the relationship between epigenetic markers (such as DNA methylation or histone modifications) and seed longevity remains an area of emerging research. While numerous associations have been identified in agricultural species, direct causal evidence is still limited.

**Table 1:** Candidate genes regulating seed longevity and deterioration.

Candidate Genes	Function	Plant	References
*HSF9A*	Controls the process of seed aging	*Medicago truncatula*	[34]
*ABI3*	Activation of *HSFA9* expression and conferral of desiccation tolerance	*Medicago truncatula*	[30]
*LEC1*	Affect multiple processes of maturation and consequently seed longevity	*Arabidopsis*	[108]
*ABI5*	Regulation of RFOs and LEAs levels, as well as the expression of photosynthesis-related nuclear genes	*Arabidopsis*	[92]
*HIRA*	Reduction in histone H3 levels leading to shortened seed life	*Arabidopsis*	[105]
*OGG1*	DNA damage repair	*Arabidopsis*	[42]
*RSL1*	Enhance GA-induced response dynamics	*Arabidopsis*	[109]
*TIP3*	Prolonged seed longevity via ABA pathway mediation	*Arabidopsis*	[109]
*ASPG1*	Seed longevity, dormancy, and germination through mechanisms involving SSP degradation and GA signaling modulation	*Arabidopsis*	[109]
*VTE1* *VTE2*	Regulation of tocopherol biosynthesis	*Arabidopsis*	[110]
*RAP2-12*	Control oxidative stress situations	*Arabidopsis*	[111]
*Rc*	Results in accumulation of proanthocyanidins	Rice (*Oryza sativa* L.)	[112]
*bZIP23* *PER1A*	Clear ROS	Rice (*Oryza sativa* L.)	[113]
*miR156*	Regulation of *IPA1* expression to inhibit the GA biosynthesis pathway, enhancing seed dormancy and resistance to deterioration	Rice (*Oryza sativa* L.)	[114]
*miR164* *miR6260* *miR5929*	Activation of stress responses and the antioxidant system	Orange (*Citrus sinensis* L.)	[107]
*α-GAL*, *RAFS*	Provide energy and reduce excessive reactive oxygen species	Maize (*Zea mays* L.)	[115]
*ZmAGA1*	Hydrolyzing RFOs as well as a precursor, galactinol	Maize (*Zea mays* L.)	[116]
*LOX*	Modulate lipid peroxidation	Maize (*Zea mays* L.)	[117]
*P5CS1*	Catalyzed Proline Biosynthesis	Oat (*Avena sativa* L.)	[118]
*AsDMP1* *AsDMP19*	Maintain redox steady state	Oat (*Avena sativa* L.)	[119]
*AMY1, BMY1 CTR1, NPR1*	Regulation of CAT, SOD, and GPx activities	Onion (*Allium cepa* L.)	[75]
*GolS1_A, GolS2_B*	RFOs Biosynthesis	Soybean (*Glycine max* L.)	[120]
*RS1, RS3*	RFOs Biosynthesis	Soybean (*Glycine max* L.)	[121]
*RS2_B*	RFOs Biosynthesis	Soybean (*Glycine max* L.)	[122]
*SS*	Stachyose Biosynthesis	Soybean (*Glycine max* L.)	[123]
*RS2*	RFOs and Stachyose Biosynthesis	Soybean (*Glycine max* L.)	[124]
*MDH*	Organic acid synthesis	*Carthamus tinctorius* L.	[125]
*Clpb1* *Clpb4*	Regulating Chaperonins	Apple (*Malus domestica Borkh.*)	[126]
*PLD*	Membrane lipid degradation and damage	Dawn Redwood (*Metasequoia glyptostroboides* Hu.)	[127]
*BnLIP1*	Participate in lipid metabolism	Cabbage (*Brassica napus* L.)	[128]
*SSIIIb*	Starch synthesis	Wheat (*Triticum aestivum* L.)	[129]
*PsAKR1*	Detoxifies reactive carbonyl compounds	Tobacco (*Nicotiana tabacum* L.)	[130]
*AhSOD*	Clear ROS	Peanut (*Arachis hypogaea* L.)	[131]

## 6. Seed Priming Enhances the Vigor of Deteriorated Seeds

The storage of orthodox seeds under low-temperature, dry, and oxygen-free conditions can effectively delay seed deterioration, while seed priming technology can restore the vigor of aged seeds. Seed priming enables seeds to complete preparatory processes for germination, such as enzyme activation, DNA repair, and membrane system restoration, promoting rapid and synchronized germination under favorable conditions. This enhances seedling emergence and establishment rates [132].

### 6.1. The Principle of Seed Priming Technology

**Figure 2:**
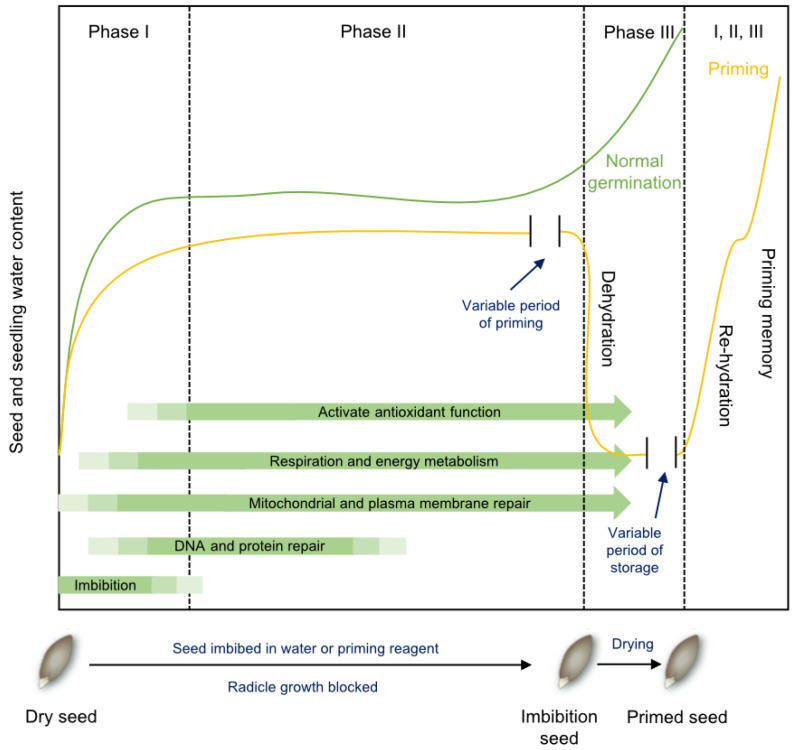
The principle of seed priming to enhance germination (Modified from [16]). Seeds placed in water progress through three phases of imbibition, including imbibition (Phase I), lag phase (Phase II), and radicle emergence and growth (Phase III). During priming, water uptake is restricted by imbibition in osmotic solutions or by limiting the amount of water provided. This extends Phase II while preventing seeds from entering Phase III. Seeds retain desiccation tolerance and can be dried, stored, and distributed for planting. When subsequently imbibed, the priming treatment shortens Phase II, resulting in a faster transition to Phase III and germination completion.

Seed priming, also referred to as controlled prehydration, involves hydrating seeds to advance germination-related metabolic processes followed by dehydration before sowing. This technique exploits the relationship between imbibition dynamics and water potential regulation. By using osmotic solutes or restricting water availability, radicle protrusion is prevented. However, critical metabolic activities during germination Phase II—such as DNA repair, protein turnover, membrane system restoration, antioxidant system activation, and enhanced respiratory and energy metabolism—continue to progress (Figure 2) [16]. Importantly, DNA synthesis, cell division, and embryonic expansion remain arrested, preserving desiccation tolerance and allowing primed seeds to be dehydrated for storage and planting.

As shown in Figure 2, Phase II of imbibition can be extended by reducing water potential. Generally, seeds retain desiccation tolerance if radicle emergence is suppressed, enabling safe post-priming dehydration. However, prolonged priming (over-priming) may cause radicle tip damage and subsequent poor seedling growth. After storage, primed seeds exhibit accelerated water uptake under adequate moisture, shortening Phase II duration and facilitating rapid radicle emergence and growth, thereby significantly reducing the time from sowing to seedling establishment [8,16].

Seed priming is a complex process whose efficacy is influenced by multiple factors, including the osmotic potential of the priming environment, priming duration, temperature, aeration conditions, light exposure, seed quality, as well as post-priming re-drying protocols and storage conditions [133]. These factors often interact synergistically or antagonistically. In liquid priming, precise control of osmotic potential is critical. An optimal osmotic potential allows seeds to achieve moderate hydration without triggering visible germination (i.e., radicle protrusion). Post-priming re-drying conditions also significantly affect priming outcomes. After priming, gradual re-drying methods are generally employed to avoid rapid dehydration, which could otherwise shorten seed longevity. The hydration–dehydration cycling method has proven effective in maintaining priming benefits by minimizing desiccation stress and preserving seed viability.

### 6.2. Vigor-Enhancing Priming Technologies for Deteriorated Seeds

Current seed priming methodologies predominantly include hydro-priming, osmo-priming, chemo-priming, solid-matrix priming, phytohormonal priming, bio-priming, nutrition-priming, nano-priming, and physical priming. Each method offers specific advantages: Water priming is cost-effective and pollution-free, though it may result in uneven germination; Osmotic priming effectively prevents premature germination in highly deteriorated seeds; Solid-matrix priming controls the growth environment while providing nutrients; Plant hormone priming enhances antioxidant defenses, surpassing water priming in repair efficacy; Biological priming utilizes microbial metabolites to repair damaged membranes, maintain integrity, and reduce electrolyte leakage; Nano-priming employs nanoparticles to penetrate seed coats, deliver trace elements to embryos, neutralize ROS through surface-active sites, reduce lipid peroxidation, and activate antioxidant systems. These techniques have been applied to rejuvenate aged seeds in crops such as wheat, maize, soybean, and onion [133,134,135,136]. Table 2 summarizes recent advancements in seed priming for vigor restoration. Notably, the effectiveness of these techniques is highly dependent on the species, degree of deterioration, and environmental conditions.

Recent advancements in seed priming technologies have revolutionized seed enhancement strategies, offering precision solutions to improve germination efficiency, stress resilience, and crop productivity. Nano-priming, utilizing engineered nanoparticles (e.g., ZnO, TiO_2_, and chitosan-based NPs), enables targeted delivery of micronutrients and reactive oxygen species (ROS) scavenging. For instance, tomato seeds treated with 50 ppm TiO_2_ nanoparticles exhibited 32% faster germination under saline conditions by enhancing aquaporin-mediated water uptake and antioxidant enzyme activity [137]. Bio-priming, which integrates beneficial microbes like the novel strain SH-8, activates systemic resistance and nutrient solubilization, improving drought tolerance by 20% in wheat [138]. Hybrid approaches such as physio-chemical priming combine cold plasma treatment with phytohormones (e.g., salicylic acid) in rice, enhancing plant growth and uptake of the nutrients under salinity stress while decreasing ROS production by increasing antioxidant enzyme activities [139].

Future trends emphasize multi-modal priming systems that synergize nanotechnology, biotechnology, and data-driven precision. Smart hydrogel-based priming with stimuli-responsive materials (e.g., pH/temperature-sensitive cellulose matrices) enables controlled release of growth regulators and real-time moisture monitoring, enhancing storage stability. In rice, Song et al. identified and genetically confirmed that DNA hypomethylation in the *acquired cold tolerance 1* (*ACT1*) gene, induced by multigenerational cold stress, promotes both cold tolerance acquisition and its stable inheritance [140]. Although this study did not employ priming treatments, the findings imply that epigenetic modifications induced by priming could be stably inherited by subsequent seed generations. Therefore, epigenetic priming (e.g., targeted DNA methylation or histone acetylation) may represent a novel breakthrough in seed priming technologies, enabling heritable enhancements in stress tolerance and longevity. CRISPR-mediated epigenetic priming, using transient gene activation via dCas9 systems, promises non-transgenic enhancement of stress tolerance genes (*OsDREB2A*, *AtHSFA2*) [141,142]. Additionally, green priming technologies focusing on eco-friendly nanoparticles (biosynthesized NPs) and biodegradable carriers are gaining traction to address environmental concerns.

**Table 2:** Priming techniques for rejuvenating deteriorated seed vigor documented in recent scientific investigations.

Method	Agent	Description	Crop	Aging Test	References
Hydro-priming	Water	Priming 10 h, improve germination rate	Napa cabbage (*Brassica rapa var. glabra Regel*)	NA * (3 years)	[143]
		Priming 12 h, improve germination rate	Maize (*Zea mays* L.)	AA * (57 °C, 24 h)	[134]
Osmo-priming	PEG	Priming 3 days, improve antioxidant enzyme activity and germination rate	*Pinus thunbergii*	AA	[41]
	KNO_3_	Priming 12 h, improve seedling length, dry weight, and germination rate	Milk Thistle (*Silybum marianum* (L.) Gaertn)	AA (45 °C, 2 days)	[144]
	CaCl_2_	Priming 6 h, improve antioxidant enzyme activity and germination rate	Safflower (*Carthamus tinctorius* L.)	AA (43 ± 1 °C, 100% RH *, 12 h)	[145]
Hormone priming	GA	Priming 12 h, improve germination rate	Maize (*Zea mays* L.)	AA (45 °C, 95% RH for 4, 7 days)	[12]
		Improve germination rate, promote seedling growth	Wheat (*Triticum aestivum* L.)	AA (40 °C, 100% RH, 72 h)	[133]
	SA	Priming 12 h, improved seed quality	Lentil (*Lablab purpureus* L.)	AA (35 °C, 90% RH, 2 days)	[7]
		Priming 8 h, increasing enzyme activity and reducing malondialdehyde (MDA) content	Soybean (*Glycine max* L.)	AA (40 °C, 100% RH, 2 days)	[146]
Solid matrix priming	Vermiculite	Priming 36 h, improve antioxidant enzymes and seed quality	Bitter gourd (*Momordica charantia* L.)	AA (40 °C, 100% RH for 3, 6, 9 days)	[147]
		Priming 16 h, improved the germination rate	Cabbage (*Brassica oleracea var. capitata* L.)	NA	[148]
	Micro-Cel E	Priming 5 days, improve germination rate and decreased mean germination time	Switchgrass (*Panicum virgatum* L.)	AA (42 °C, 95% RH for 10, 20 days)	[149]
Bio-priming	Azotobacter	Priming 16 h, improve germination rate and vigor	Onion (*Allium cepa* L.)	NA (1, 2, 3 year)	[136]
	Bacillus megaterium	Priming 5 h, improve shoot length, root length, and seedling vigor index	Soybean (*Glycine max* L.)	AA (42 °C, 95% RH, 72 h)	[150]
Caffeic acid	Caffeic acid	Priming 6 h, improve germination rate	Soybean (*Glycine max* L.)	AA (45 °C, 95% RH, 24 h)	[135]
Melatonin	Melatonin	Priming 24 h, improve antioxidant enzymes	Oat (*Avena sativa* L.)	NA (48 days)	[118]
Spermidine	Spermidine	Priming 24 h, improve antioxidant enzymes activity and GA content	Rice (*Oryza sativa* L.)	AA (43 °C, 98% RH, 4 days)	[151]
Nano	AgNPs	Priming 24 h, improve germination rate	Rice (*Oryza sativa* L.)	NA (25–30 °C, 3 years)	[11]
		Priming 24 h, increased the total phenolic content of seedlings and CAT activity	Broad bean (*Vicia faba* L.)	NA (5 ± 1.5 °C, 50% RH, 7 years)	[152]
Plant-derived smoke	Karrikinolide	Priming 16 h, improve germination rate, fresh and dry weight	Marrow (*Cucurbita pepo* L.), Cabbage (*Brassica oleracea var. capitata* L.) and Pepper (*Piper nigrum* L.)	AA (40 °C,100% RH, 48 h)	[153]
Union priming	PEG+GA_3_, PEG+ABA	Priming 6 h, improve germination rate	Moench (*Abelmoschus esculentus* L.)	AA (40 ± 10 °C, 100% RH, 72 h)	[154]
	KH_2_PO_4_ + KNO_3_	Priming 12 h, promote germination	Muskmelon (*Cucumis sativus* L.)	NA (2 years)	[155]
Physical priming	HVEF (High-voltage electrostatic field)	Priming 55 min, improve antioxidant enzyme activity, reduce leakage rate	Rice (*Oryza sativa* L.)	AA (40 °C, 100% RH, 8 days)	[156]
	PAW (Plasma activated water)	Improve germination rate	Pepper (*Capsicum annuum* L.)	NA (2 years)	[157]

* NA means natural aged; AA means accelerated aged; RH means relative humidity.

### 6.3. Mechanisms Underlying Seed Priming-Mediated Enhancement of Vigor in Deteriorated Seeds

**Figure 3:**
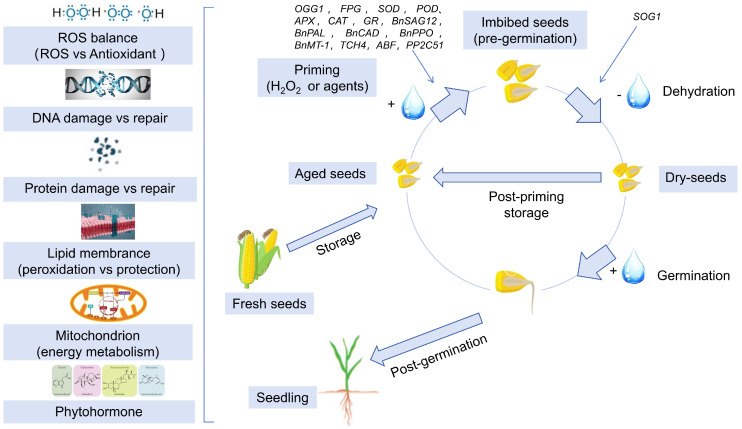
Mechanisms of seed priming in enhancing the vigor of aged seeds. Seed priming mechanism: During storage, seeds undergo deterioration primarily through oxidative damage to DNA, proteins, and lipids. Priming treatments counteract these effects by enhancing antioxidant defenses, activating repair systems, and ultimately restoring germination vigor.

Primed seeds exhibit significantly enhanced vigor, improved seedling emergence and establishment rates, and elevated stress tolerance. However, the underlying mechanisms of priming are not fully understood. The relevant literature on seed priming mechanisms reveals two predominant pathways: (1) initiation of germination-associated preparatory processes that facilitate the transition from a quiescent desiccated state to a full-germination state, thereby augmenting germination potential; (2) imposition of controlled abiotic stress during priming, which activates stress-responsive signaling cascades and induces systemic tolerance enhancement (Figure 3).

During seed priming, numerous enzymes associated with the metabolism of storage reserves (carbohydrates, proteins, and lipids) are activated, such as amylases, proteases, and isocitrate lyase, thereby promoting the mobilization of reserves [158,159]. The activity of α-amylase, which facilitates starch hydrolysis, is positively correlated with soluble sugar content during germination. Comparative studies demonstrate that hydro-primed deteriorated wheat (*Triticum aestivum*) seeds exhibit significantly enhanced α-amylase activity, soluble sugars, and soluble protein levels compared to non-primed controls [160].

Seeds scavenge excess intracellular ROS through antioxidant enzymes and non-enzymatic antioxidants to counteract oxidative stress and enhance viability. The antioxidant function of seeds is activated during priming treatments, thereby improving germination rates, and increasing seedling vigor at sowing [132]. Ulhassan et al. applied silicon nanoparticle (SiNP) priming treatment to *Brassica napus* seeds, which upregulated the expression of oxidative stress-related genes (*BnPAL*, *BnCAD*, *BnPPO*, *BnMT-1*) and chlorophyll synthesis-related genes (*BnCAO*, *BnCHLG*, *BnPOR*), while suppressing the senescence-associated gene *BnSAG12*, thereby enhancing resistance to the deterioration [161]. Polyethylene glycol-6000 (PEG-6000) priming of soybean seeds significantly upregulated antioxidant enzyme-encoding genes (*GST*, *SOD*, *POD*), reduced intracellular ROS levels, and improved germination and seedling establishment rates in deteriorated seeds [162]. Thioredoxin (TRX), a conserved protein in plant oxidative stress responses, directly scavenges H_2_O_2_ and ROS radicals [163]. Melatonin priming of aged oat (*Avena sativa*) seeds elevated intracellular antioxidants TRX and GST, improving germination rates [118].

DNA repair is a critical event in seed germination, initiated during the seed imbibition process. Following the hydro-priming treatment of eggplant (*Solanum melongena*) seeds, the expression levels of key DNA repair-related genes *OGG1* and *FPG* were upregulated to facilitate DNA repair during germination [164]. In water-treated *Medicago truncatula* seeds during redrying, the SOG1 gene—encoding a key regulatory factor in plant DNA damage repair—exhibited significantly elevated expression [165]. As DNA repair serves as a critical rate-limiting step during DNA replication, primed cabbage (*Brassica oleracea*) seeds displayed higher DNA synthesis rates and elevated cell division levels, demonstrating that priming enhances DNA repair processes [166,167].

Damaged proteins caused by dehydration–rehydration cycles require repair or removal. It has been reported that proteins involved in the translation process are primary targets of seed protection and repair mechanisms. Except for those integral to the translational machinery, other proteins may be removed or replaced in imbibing seeds [168]. Translation-associated proteins, such as ribosomal proteins, can reassemble ribosomes to initiate the synthesis of new proteins (replacing oxidatively damaged ones), making these proteins essential for sustaining translation. Meanwhile, LEAs rapidly accumulate during early priming stages, reducing further protein damage through physical shielding and antioxidant activity, thereby stabilizing cellular homeostasis [169,170]. LEAs have been reported to protect proteins from dehydration-induced damage, and their accumulation has been observed in primed sugar beet (*Beta vulgaris*) [171] and rapeseed (*Brassica napus*) seeds, facilitating repair of dehydration-related injuries [172]. Beyond translation-associated proteins, other critical proteins also require repair or removal. Silver nanoparticle (AgNP) priming of deteriorated rice (*Oryza sativa*) seeds triggered the restoration of functional proteomes by upregulating aquaporin genes *PIP1;1* and *PIP2;1* [11]. L-isoaspartyl methyltransferase, a repair enzyme activated under oxidative stress in seeds, plays a vital role in repairing proteins involved in ribosomal RNA (rRNA) processing within the nucleus [173,174]. Osmopriming of deteriorated tomato (*Solanum lycopersicum*) seeds restored L-isoaspartyl methyltransferase activity, confirming its role in repairing damaged cellular proteins during priming [171].

The regulation of plant hormone signaling is a central mechanism in seed priming. This process involves promoting the expression of gibberellin (GA)- and cytokinin (CTK)-related genes while reducing ABA levels, thereby modulating the ABA-GA balance [147]. In polyethylene glycol-6000 (PEG-6000)-primed deteriorated soybean (*Glycine max*) seeds, the expression of seven IAA-coding genes (e.g., *Glyma.02G142500*, *Glyma.03G158700*) was upregulated. Additionally, *Glyma.13G095200* (encoding *TCH4*, a brassinosteroid (BR)-responsive transcription factor within the xyloglucan endotransglucosylase/hydrolase (XTH) family that regulates BR- and IAA-mediated cell elongation) exhibited upregulated expression. Conversely, *Glyma.14G162100* (encoding PP2C51, a component of the ABA signaling pathway) and *Glyma.10G071700*/*Glyma.19G194500* (encoding ABA-responsive element-binding factors, ABFs) showed downregulated expression [162]. This treatment enhanced BR and IAA signaling pathways while suppressing ABA signaling, thereby antagonizing ABA-mediated inhibition, and promoting germination in aged seeds. In a study where deteriorated wheat seeds were primed with GA, cell membrane integrity improved, leading to reduced electrolyte leakage. Concurrently, proline and soluble sugar levels increased, while MDA content decreased [133].

## 7. Conclusions and Future Outlook

Seed deterioration is a complex process influenced by both environmental factors and genetic regulation. Understanding its mechanisms is critical for enhancing seed viability and facilitating long-term conservation of germplasm resources. Recent advances in multi-omics technologies (e.g., genomics, transcriptomics, metabolomics, proteomics, epigenomics) and gene-editing tools (e.g., CRISPR/Cas9) have revolutionized mechanistic studies of seed deterioration. Integrated approaches, including genome-wide association studies (GWAS), enable the identification of key regulatory genes, mapping of deterioration-related networks, and clarification of molecular pathways driving seed aging. There is an urgent need for functional validation of molecular mechanisms in non-model species.

Current research reveals that oxidative damage to genetic material, proteins, and membrane systems constitutes the core mechanisms driving seed deterioration. Such damage disrupts cellular functions or induces cell death in embryo cells, ultimately leading to the loss of germination capacity. However, seeds possess intrinsic anti-deterioration mechanisms, including antioxidant systems (e.g., enzymatic scavengers such as SOD and CAT), detoxification systems (e.g., GSTs), and repair systems (e.g., DNA ligases and chaperone proteins), which collectively delay the deterioration process. Seed priming treatments counteract aging effects by reactivating these defense responses, thereby restoring vigor and enhancing germination capacity in aged seeds.

To achieve long-term germplasm conservation and sustainable agricultural productivity, genetic improvement and seed treatment innovations are complementary strategies. Future research should focus on employing genomic/genetic engineering strategies to identify critical regulatory genes for breeding deterioration-resistant crop varieties with enhanced storage tolerance, developing optimized storage protocols and precision seed priming methods based on seed deterioration mechanisms to delay deterioration processes and extend seed longevity, and addressing the challenges in scaling up priming technologies for large-scale agricultural applications. Through these approaches, postharvest economic losses caused by seed deterioration can be significantly reduced, thereby safeguarding global food security.
